# Iterative Bayesian Model Averaging: a method for the application of survival analysis to high-dimensional microarray data

**DOI:** 10.1186/1471-2105-10-72

**Published:** 2009-02-26

**Authors:** Amalia Annest, Roger E Bumgarner, Adrian E Raftery, Ka Yee Yeung

**Affiliations:** 1 Institute of Technology/Computing and Software Systems, Box 358426, University of Washington, Tacoma, WA 98402, USA; 2 Department of Microbiology, Box 358070, University of Washington, Seattle, WA 98195, USA; 3 Department of Statistics, Box 354320, University of Washington, Seattle, WA 98195, USA

## Abstract

**Background:**

Microarray technology is increasingly used to identify potential biomarkers for cancer prognostics and diagnostics. Previously, we have developed the iterative Bayesian Model Averaging (BMA) algorithm for use in classification. Here, we extend the iterative BMA algorithm for application to survival analysis on high-dimensional microarray data. The main goal in applying survival analysis to microarray data is to determine a highly predictive model of patients' time to event (such as death, relapse, or metastasis) using a small number of selected genes. Our multivariate procedure combines the effectiveness of multiple contending models by calculating the weighted average of their posterior probability distributions. Our results demonstrate that our iterative BMA algorithm for survival analysis achieves high prediction accuracy while consistently selecting a small and cost-effective number of predictor genes.

**Results:**

We applied the iterative BMA algorithm to two cancer datasets: breast cancer and diffuse large B-cell lymphoma (DLBCL) data. On the breast cancer data, the algorithm selected a total of 15 predictor genes across 84 contending models from the training data. The maximum likelihood estimates of the selected genes and the posterior probabilities of the selected models from the training data were used to divide patients in the test (or validation) dataset into high- and low-risk categories. Using the genes and models determined from the training data, we assigned patients from the test data into highly distinct risk groups (as indicated by a p-value of 7.26e-05 from the log-rank test). Moreover, we achieved comparable results using only the 5 top selected genes with 100% posterior probabilities. On the DLBCL data, our iterative BMA procedure selected a total of 25 genes across 3 contending models from the training data. Once again, we assigned the patients in the validation set to significantly distinct risk groups (p-value = 0.00139).

**Conclusion:**

The strength of the iterative BMA algorithm for survival analysis lies in its ability to account for model uncertainty. The results from this study demonstrate that our procedure selects a small number of genes while eclipsing other methods in predictive performance, making it a highly accurate and cost-effective prognostic tool in the clinical setting.

## Background

### Introduction and Previous Work

Until recently, oncologists relied primarily on tumor stage and morphology to help outline an appropriate course of treatment for their cancer patients. Malignant tumors were generally resected in operable cases, and follow-up radiation therapy was provided to victims exhibiting advanced-stage diseases. This methodology proved problematic in that a number of low-risk patients experienced cancer recurrence or death within a short time frame, while a contingent of high-risk patients went into permanent remission despite the bleak nature of their original prognoses. This indicated a need to explore other indicators by which doctors could understand the underlying prognosis of a given disease and decide on a treatment plan that would optimize the patient's chances for survival.

Microarray technology provides a promising avenue. The availability of thousands of gene expression levels has enabled the pursuit of a new direction in cancer research. In particular, gene expression patterns can be thought of as multidimensional quantitative "expression phenotypes" which can in turn be correlated with clinical outcome. Because a single microarray can measure the expression levels of tens of thousands of genes simultaneously, the challenge lies in the development of data mining methods and tools to extract biological meaning from this immense amount of data. More specifically, the aim is to filter the expression dataset down to the smallest possible subset of accurate predictor genes. Reducing the number of predictor genes both decreases clinical costs and mitigates the possibility of overfitting due to high inter-variable correlations [1].

The most common approach to identify a manageable group of predictor genes is called feature selection, in which a subset of relevant "features" (or variables) is selected from the full dataset in order to produce a robust learning model [2,3]. A well-designed feature selection algorithm will choose a small set of variables that is highly predictive of clinical outcome. Univariate feature selection methods evaluate the usefulness of each variable on an individual basis. Examples of univariate techniques include the t-test [4], the signal-to-noise ratio [5], the Cox proportional hazards model [6], threshold number of misclassification (TNoM) score [7], the between-groups to within-groups sum of squares (BSS/WSS) ratio [8], and mean aggregate relevance [9]. Multivariate methods are more sophisticated in that they perform combinatorial searches within the feature subspace to evaluate the effectiveness of groups of genes. Examples include Recursive Feature Elimination (RFE) [10], genetic algorithms [11-13], floating search [14], and top-scoring pair methods [15,16]. Despite some evidence to the contrary (e.g., [17]), multivariate selection algorithms are generally preferable to univariate ones because they cut down on dependencies between variables and often lead to models with fewer predictive variables [18,19]. However, selecting multivariate features from microarray data is non-trivial since the number of patient samples is often limited (usually under a hundred) and the number of genes is large (usually tens of thousands).

Subsequent to or concurrent with the feature selection process, a supervised machine learning technique can be applied to generate a predictive function using the selected variables from a set of training data [20,21]. In a supervised learning algorithm, the input is a set of training samples paired with the corresponding labels of those samples. If the labels are exhaustive discrete classes to which the samples belong (e.g. "survived beyond five years" and "died before five years"), then the learning model is a *classifier *(for a review of classification techniques in supervised machine learning, see [22]). With microarray data, the most common approach is to apply a classification algorithm in which the patients are split into subcategories corresponding to different prognoses or diagnoses. In general, the subcategories are static and based on thresholds associated with some clinical variable (e.g., time to metastases). Classification studies on microarray data have used gene expression levels to distinguish diseased tissue samples from normal ones [23,24], identify cancer subtypes [5,25], and assign discrete risk groups for survival prognosis [13,19,26-28]. See Hu et al. [29] for a comparative analysis of classification methods for microarray data. Depending on whether the classifier is used to select relevant features, feature selection methods can also be divided into filter and wrapper methods [30]. Wrapper methods utilize the classifiers as evaluation functions and search for the optimal gene set for classification. In contrast, filter methods rely on general characteristics of the training data to select genes without involving any classifier for evaluation. Many filter methods evaluate a gene based on its discriminative power for the target classes without considering its correlations with other genes. Wrapper algorithms can perform better than filter algorithms, but they typically require orders of magnitude more computation time.

In cancer research, gene expression data is often reported in tandem with time to event information (such as time to metastasis, death, or relapse). In order to take advantage of these continuous clinical variables under a supervised framework, survival analysis can be applied. Survival analysis on microarray data differs from classification in that the sample labels are continuous rather than discrete. The overall goal in survival analysis research is to create the strongest predictive model of patient survival, and the most important components of this process are feature selection and model construction. In the context of survival analysis, a *model *refers to a set of selected genes whose regression coefficients have been calculated for use in predicting survival prognosis [31]. In the application of survival analysis to high-dimensional microarray data, a feature selection algorithm identifies this subset of genes from the gene expression training dataset. These genes are then used to build a statistical model for the continuous time to event data [32]. The choice of feature selection algorithm determines which genes are chosen and the number of predictor genes deemed to be relevant, whereas the statistical model gives the distribution of the time to the event.

In recent years, a number of studies have applied survival analysis to microarray data. Beer et al. [33] used univariate Cox proportional hazards regression along with leave-one-out cross validation on an 86-sample lung cancer dataset to develop a risk index based on 50 genes that successfully divided an independent test set of patients into high- and low-risk groups. Lu et al. [34] improved on these results by using multivariate Cox proportional hazards model with bootstrap resampling and forward selection to obtain a 64-gene model that yielded a greater predictive accuracy than Beer et al. A popular approach to deal with high dimensionality in survival analysis is dimension reduction. For example, Bair and Tibshirani [35] proposed a semi-supervised version of principal components analysis that is capable of generating a continuous predictor of patient survival. Their algorithm consistently selected fewer than 20 genes and successfully divided patients into high- and low-risk groups in four different cancer subtypes: lymphoma, breast cancer, lung cancer, and acute myeloid leukemia. Partial least squares (PLS) reduces the dimension of the original variables by constructing a smaller collection of latent variables that are linear combinations of the original variables. The application of PLS in conjunction with the Cox proportional hazards model in survival analysis to microarray data has been investigated [36,37]. A drawback of dimension reduction techniques is that usually a relatively large number of genes (variables) are selected in the reduced dimension space. Penalized methods such as LASSO (least absolute shrinkage and selection operator) [38,39] is a variable selection method, and hence is an alternative to dimension reduction techniques as it can be used when the number of samples is smaller than the number of variables (genes). Zhang et al. [40] studied the theoretical properties of an adaptive LASSO method for the Cox proportional hazards model. Kaderali et al. [41] proposed a multivariate Cox regression model embedded in a Bayesian framework that combines dimension reduction and regression in one single step. They used a hierarchical prior distribution that is strongly peaked around zero on the regression parameters so as to produce a small number of relevant genes with non-zero regression parameters. A distinctive characteristic in Kaderali et al. is that they assume that the constant baseline hazard rate in the Cox proportional hazards model is known, and aim to directly predict survival times of patients. Recently, Bovelstad et al. [42] compared the prediction performance of seven methods that are based on the Cox proportional hazards model over three microarray datasets, and showed that ridge regression has the overall best performance. In their empirical studies, Bovelstad et al. focused on prediction accuracy instead of the number of selected genes.

The accelerated failure time (AFT) model [43] is a linear regression model in which the response variable is the logarithm or a known monotone transformation of event times. Unlike the Cox proportional hazards model which assumes that the ratio of the hazard functions does not depend on time and the baseline hazard is unspecified, one can directly predict event times using the AFT model and hence, is a useful alternative to the Cox model. However, the AFT model has not been widely used in practice due to difficulties in computing the regression parameters even when the number of variables is small. Recently, Huang and colleagues [44,45] studied the use of penalized methods in the AFT model for survival analysis. However, these proposed methods were not applied to microarray datasets in which thousands or tens of thousands of variables are available. Datta et al. [46] investigated the performances of LASSO and PLS on microarray data using the AFT model. They showed that LASSO performed better than PLS when there are many noise variables in their simulation studies.

### Our Contributions

A problem with most feature selection algorithms used to produce continuous predictors of patient survival is that they fail to account for model uncertainty. With thousands of genes and only tens to hundreds of samples, it often happens that a number of different models describe the data about equally well. In this paper, we apply the Bayesian Model Averaging (BMA) method [47,48] to select a subset of genes for survival analysis on microarray data. Instead of choosing a single model and proceeding as if the data were actually generated from it, BMA combines the effectiveness of multiple models by taking the weighted average of their posterior distributions. In addition, BMA consistently identifies a small number of predictive genes [19,31], and the posterior probabilities of the selected genes and models are available to facilitate an easily interpretable summary of the output. Yeung et al. [19] extended the BMA algorithm to classify high-dimensional microarray; they dealt with the very large number of potential predictors using an iterative approach. Here we further extend their iterative BMA method to survival analysis. In particular, we developed and implemented the iterative BMA method for survival analysis as a Bioconductor package, and we also demonstrated our algorithm on two cancer datasets. Our results reveal that iterative BMA consistently selects a small number of predictor genes while providing greater predictive accuracy than other algorithms, and the models themselves are simple and amenable to biological interpretation.

## Methods

### Data

#### Breast Cancer

The first dataset in this study consists of patient samples from primary invasive breast carcinomas [27,49]. The breast cancer dataset from van't Veer et al. was comprised of 78 training samples and 19 test samples. Previously van't Veer et al. identified a 70-gene predictive signature which classified patient samples into good versus poor prognosis groups. Subsequently, van de Vijver et al. [49] acquired a test set of 295 patient samples with clinical data on which to validate the 70-gene predictive signature. Of these 295 patient samples, 61 samples overlapped with the 78 training samples from van't Veer et al. Since different clinical data and survival information were made available from these two publications, we used these 61 overlapping samples as our training set and the remaining 234 samples as our test set, both of which are available on our supplemental website . The samples in both breast cancer datasets were hybridized to two-color microarrays containing approximately 25,000 genes. Previously, Yeung et al. [19] filtered the van't Veer et al. dataset down to 4919 significantly regulated genes (at least a 2-fold difference and p-value < 0.01 in at least three samples), and we have chosen to conduct our analysis with these 4919 genes. Of the 295 total samples in our training and validation datasets, the times to death or censoring ranged from 0.05 to 18.3 years, with a median of 7.2 years. 216 patients (73%) were still alive at the final follow-up visit. See Table 1 for a summary of the breast cancer data.

**Table 1:** Summary of Breast Cancer and DLBCL Datasets

Dataset	Total Number of Samples	# Training Samples	# Validation Samples	Number of Genes
Breast Cancer	295	61	234	4919
DLBCL	240	160	80	7399

#### Lymphoma

Our second dataset consists of tumor samples from 240 patients diagnosed with diffuse large B-Cell lymphoma (DLBCL) [50]. Roughly 60% of DLBCL victims who are treated with chemotherapy do not survive, and the disease comprises 30–40% of all non-Hodgkin lymphomas [51,52]. This DLBCL dataset was generated and first analyzed by Rosenwald et al. [50], and the expression profiles from 7399 genes along with corresponding patient information can be downloaded from their supplemental website . The raw data were processed with "lymphochip" cDNA microarrays [53], which are specialized to include genes that are known to be preferentially expressed within the germinal centers of lymphoid organs. Survival times ranged from 0 to 21.8 years, with a median of 2.8 years across all samples. Of the 240 patients, only 102 (42.5%) were still alive at the final follow-up visit. Rosenwald et al. randomly divided the dataset into 160 training samples and 80 validation samples, and we have chosen to preserve their division in order to allow a direct comparison of results. See Table 1 for a summary of the breast cancer and DLBCL datasets.

### Bayesian Model Averaging (BMA)

The strength of BMA lies in its ability to account for model uncertainty, an aspect of analysis that is largely ignored by traditional stepwise selection procedures [47]. These traditional methods tend to overestimate the goodness-of-fit between model and data, and the model is subsequently unable to retain its predictive power when applied to independent datasets [31,54]. BMA attempts to solve this problem by selecting a subset of all possible models and making statistical inferences using a weighted average of these models' posterior distributions.

The core of the BMA algorithm is depicted in Equation (1) below [47]. Let Ψ denote the quantity of interest, and let *S *= {*M*_1_, *M*_2_, ..., *M*_n_} represent the subset of models selected for inclusion in the analysis. Then the posterior probability of Ψ given the training data *TD *is the weighted average of the posterior probability of Ψ given *TD *and model *M*_i_, multiplied by the posterior probability of model *M*_i _given *TD*. Summing over all the models in set *S*, we get:

(1) $$\mathrm{Pr}(\Psi |TD)=\sum_{i\in S}\mathrm{Pr}(\Psi |TD,M_{i})\cdot \mathrm{Pr}(M_{i}|TD).$$

There are three issues to consider before Equation (1) can be applied: obtaining the subset *S *of models to be included, estimating the value of Pr(Ψ | *TD*, *M*_i_), and estimating the value of Pr(*M*_i _| *TD*). The remainder of this subsection will address these issues.

One challenge with BMA is the sheer number of models that could potentially be explored by the algorithm, especially when dealing with microarray data. If there are *G *candidate explanatory genes in the expression set, then there are 2^G ^possible models to consider. When working with tens of thousands of genes, such an undertaking is computationally intractable. In order to discard the noncontributory models and obtain a subset that approximates an average over all 2^G ^possibilities, Raftery [47] proposed to use the regression by leaps and bounds algorithm from Furnival and Wilson [55]. This algorithm takes a user-specified input "*nbest" *and efficiently returns the top *nbest *models of each size (maximum 30 variables). Following application of the leaps and bounds algorithm, the Occam's window method of Madigan and Raftery [56] can be used to reduce the set of models. After identifying the strongest model returned by the leaps and bounds algorithm, the procedure can eliminate any model whose posterior probability is below the cutoff point in relation to the best model. The cutoff point can be varied, but the default is 20; that is, a model must be at least 1/20 as likely as the strongest model in order to be retained. Once this step is complete, the remaining group of models constitutes the set *S *to be used in Equation (1).

An exact calculation of the predictive distribution Pr(Ψ | *TD*, *M*_i_) requires an integration over the vector of regression parameters *θ*_i_:

(2) Pr(Ψ | *TD*, *M*_i_) = ∫ Pr(Ψ | *θ*_i_, *TD*, *M*_i_) Pr(*θ*_i _| *TD*, *M*_i_) d*θ*_i_.

Because this integral has no closed form solution for most censored survival models, the maximum likelihood estimate (MLE) can be used as an approximation:

(3) $$\mathrm{Pr}(\Psi |TD,M_{i})\approx \mathrm{Pr}(\Psi |{\hat{\theta }}_{i},TD,M_{i}).$$

While certain techniques such as the Markov Chain Monte Carlo (MCMC) methods have been used in survival analysis to obtain a more exact predictive distribution [57], the MLE requires fewer computational resources and has been deemed sufficient for the purpose of averaging over contending models [31,58-60].

Finally, a calculation of the posterior probability of model *M*_i _given the training data *TD *involves an integral whose value is impossible to evaluate exactly. Bayes' theorem yields Equation (4), which represents the posterior probability of model *M*_i _given *TD*:

(4) Pr(*M*_i _| *TD*) ∝ Pr(*TD *| *M*_i_) Pr(*M*_i_),

where

(5) Pr(*TD *| *M*_i_) = ∫ Pr(*TD *| *θ*_i_, *M*_i_) Pr(*θ*_i _| *M*_i_) d*θ*_i_.

Pr(*TD *| *M*_i_) is the integrated likelihood of model *M*_i_, and *θ*_i _is the vector of regression parameters (*b*_0_, *b*_1_, ..., *b*_p_) of model *M*_i_. The Bayesian Information Criterion (BIC) first derived by Schwarz (1978) can be used to approximate the integral in equation (5):

(6) $$\log\mathrm{Pr}(TD|M_{i})=\log\mathrm{Pr}(TD|{\hat{\theta }}_{i},M_{i})-(k_{i}/2)\log n+O(1).$$

In equation (6), *n *represents the number of records in the data, *k*_i _is the number of regression parameters in model *M*_i_, and *O*(1) is the error term. The approximation is more accurate for many practical purposes than its O(1) error term suggests for certain reasonable choices of the prior distribution Pr(*θ*_i _| *M*_i_) [47,61]. Raftery [62] gave further empirical evidence for the accuracy of this approximation. This method is implemented in the bic.surv function that is part of the BMA R package available at 

While this section has focused on the posterior probabilities of the models included in the BMA analysis, we are also interested in obtaining the posterior probabilities for each of the individual variables (genes) involved. This information is helpful in facilitating biological discussion as it reveals which of the genes are relevant predictors. Let the expression (b_i _≠ 0) indicate that the regression parameter for gene x_*i *_exists in the vector of regression parameters *θ*_i_. Then the posterior probability that gene x_*i *_is a relevant predictor can be written as:

(7) $$\mathrm{Pr}({\text{b}}_{\text{i}}\neq 0|TD)=\sum_{{\text{M}}_{\text{S}}\text{where gene is relevant}}\mathrm{Pr}({\text{M}}_{\text{S}}|TD).$$

The posterior probability of gene x_i _is the sum of the posterior probabilities of all models in the subset *S *that include gene x_i_.

### BMA for Survival Analysis

Volinsky et al. [31] applied the Bayesian Model Averaging methods [47,48] to survival analysis. They assessed a patient's risk of stroke by using BMA to select variables in Cox Proportional Hazards models [6]. The data were made available by the Cardiovascular Health Study and included 23 variables (e.g., age, smoking history, and blood pressure) that may contribute to a patient's chances of experiencing a stroke. BMA selected a total of 5 models and 11 predictive variables, including diuretic, aspirin use, diabetes, stenosis, and timed walk. Patient risk scores were calculated by taking the weighted average of the risk scores for each of the top five contending models. The patients were then assigned to either the high-risk, medium-risk, or low-risk group based on the empirical 33^rd ^and 66^th ^percentile cutoff points in the risk scores of the training set. To assess performance, Volinsky et al. [31] created an analogue to the log-score called the partial predictive score (PPS). The PPS for BMA was compared against the PPS for the top BMA model (that is, the single model of the top five BMA models with the highest posterior probability) and against the PPS of the model returned by stepwise backward elimination. BMA exhibited the highest PPS, with a prediction mechanism 15% more effective than the top model alone and 3.5% more effective than the stepwise procedure. Furthermore, the patients assigned to a risk group using BMA experienced fewer strokes in the low-risk group and more strokes in the high-risk group when compared with the other two methods.

### Extending BMA for High-Dimensional Microarray Data

#### Iterative BMA for Classification

The BMA implementation described above is incompatible with microarray data. This is because the typical microarray dataset contains thousands or even tens of thousands of genes, but the leaps and bounds algorithm from Furnival and Wilson [55] tends to become slow when there are more than 45 variables or so. One common solution is to use stepwise backward elimination to reduce the number of genes down to 30, but this is not applicable in a situation where the number of predictive variables is greater than the number of samples. Yeung et al. [19] developed an iterative BMA algorithm that takes a rank-ordered list of genes and successively applies the traditional BMA algorithm until all genes up to a user-specified value *p *(*G*_1_, *G*_2_, ..., *G*_p_) have been processed. The authors begin by using the ratio of between-group to within-group sum of squares (BSS/WSS) [8] to rank-order the genes from the microarray dataset. As the algorithm iterates, genes with a high posterior probability (equation (7)) are retained while genes with a low posterior probability are eliminated. The default threshold for inclusion is set to 1%; genes whose posterior probabilities are less than 1% are discarded.

#### Iterative BMA for Survival Analysis

In this article, we report our efforts in extending the iterative BMA method to survival analysis, which include a number of modifications to the algorithm. First, instead of applying the BSS/WSS technique [8] to rank-order the genes in the preprocessing step, we use the Cox Proportional Hazards Model [6] to rank each individual gene. Cox regression is a popular choice in the realm of survival analysis due to its broad applicability and capacity for handling censored data. It is a semi-parametric method that quantifies the hazard rate for a subject *s *at time T as follows:

(8) *λ*(T | p_s_) = *λ*_0_(T)exp(p_s_*θ*).

In this equation, *λ*_0_(T) is the baseline hazard function at time T, p_s _is the vector of effect parameters (predictors) for subject *s*, and *θ *is the vector of unknown predictor coefficients. Cox observed that the baseline hazard function in equation (8) could be left unspecified if the effect of a covariate on one individual remains the same for all times T (e.g., if an environmental variable doubles your personal risk of dying at time 5, it also doubles your risk at time 8). Therefore, an estimation of *θ *is all that is needed. This approximation can be calculated using the partial likelihood:

(9) $$\text{PL}(\theta )={\prod_{s=1}^{n}\left(\frac{\exp(p_{s}\theta )}{\sum_{\ell \in R_{s}}\exp(p_{\ell }\theta )}\right)}^{\delta_{i}}.$$

In equation (9), R_s _is the risk set at time t_s _(where the risk set consists of individuals who have not yet experienced the event of interest), and δ_i _is an indicator for whether subject i is censored. Once the regression parameters in the Cox model are estimated by maximizing the partial likelihood, the genes can be ranked in descending order of their log likelihood.

**Figure 1:**
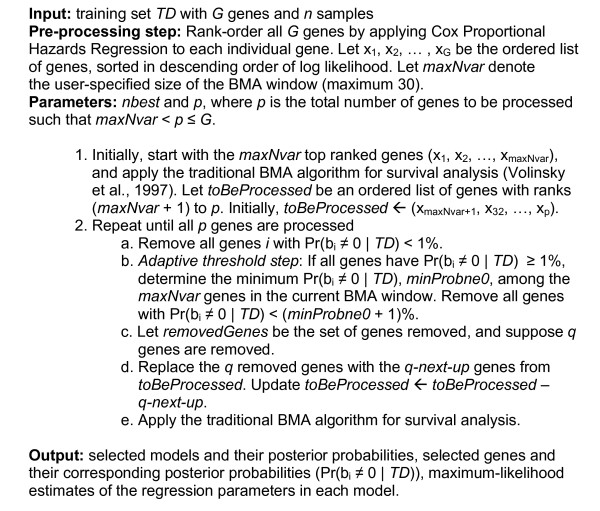
**Outline of the iterative BMA algorithm for survival analysis on microarray data**.

Following this step, the algorithm iterates through the user-specified *p *top-ranked genes, applying the traditional BMA algorithm for survival analysis [31] to each group of variables in the current BMA window (where the window size is denoted by *maxNvar*). This part of the procedure is similar to the classification method described previously; genes with high posterior probabilities are retained while genes with low posterior probabilities are eliminated. Following Yeung et al. [19], we have chosen to adopt the 1% default threshold for inclusion. The algorithm relies on the elimination of at least one gene per iteration from the current BMA window, so the method cannot proceed if all genes in the window have a posterior probability ≥ 1%. Yeung et al. proposed an "adaptive threshold" heuristic to account for this possibility, whereby the genes with the lowest posterior probabilities are removed to make room for subsequent variables. We have incorporated this heuristic into our algorithm because Yeung et al. [19] reported that its inclusion boosts predictive accuracy. See Figure 1 for an outline of the iterative BMA algorithm for survival analysis.

Furthermore, we have incorporated an additional heuristic in which the models that are discarded due to the adaptive threshold are re-considered when all the iterations are completed. Specifically, we applied the Occam's window method of Madigan and Raftery [56] to reduce the set of models remaining from the last iteration of bic.surv and the models that are discarded due to the adaptive threshold. We also re-computed the posterior probabilities of the models and the genes accordingly. In our software implementation, this heuristic is available as an option called "keepRmModels" and this option is set to FALSE by default.

### Assessment

To evaluate the performance of our method, we discretize the risk scores of patients into risk groups. The overall risk score for a single patient is the weighted average of the risk scores calculated for each model *M*_*i *_in the set *S *of contending models. The equation is as follows [31]:

(10) $$\sum_{i\in S}\left(x_{j}^{v}{\hat{\theta }}_{i})\mathrm{Pr}(M_{i}|TD\right)$$

In equation (10), ${\hat{\theta }}_{i}$ represents the vector of regression parameters for model *M*_*i *_and $x_{j}^{v}$ refers to the expression score of each gene *x*_*j *_within model *M*_*i *_for a patient in the validation dataset. Therefore, the risk score is computed by multiplying the expression scores of all genes included in model *M*_*i *_by their corresponding predictor coefficients, adding these *x*_*j*_*b*_*j *_terms together, weighing this number by the posterior probability of each model *M*_*i *_and summing over all contending models in the set *S*. Note that the predictor coefficients and the model posterior probabilities are all determined from the training data. Our implementation employs uses the user-specified "*cutPoint*" for defining high- versus low-risk groups (e.g., a *cutPoint *of 60 means the lower 60% of scores will be deemed low-risk, and the upper 40% will comprise the high-risk group) using the risk scores of patients in the training data.

The Kaplan-Meier survival curves [63], in which the proportions of surviving patients in each risk group are plotted against successive time intervals, are used to illustrate our results. An advantage of the Kaplan-Meier curve is that it takes censored data into consideration: small vertical tick-marks represent losses where patient data were censored. In addition, we measured predictive performance with the p-value calculated from the log-rank test using the central chi-square distribution. The log-rank test calculates a p-value testing the null hypothesis that the survival curves from the high- and low-risk groups are identical. Therefore, a significant p-value indicates that the two risk groups are distinct.

### Selection of Input Parameters

The main user-specified parameters to the iterative BMA algorithm for survival analysis include the number of top-ranked *p *genes to be included in the iterations, the *nbest *strongest models to be returned by the leaps and bounds algorithm from Furnival and Wilson [55], the desired *cutPoint *for separating high- from low-risk patient samples, and the size of the active BMA window (*maxNvar*). In order to determine the best combination of these input parameters, we performed a series of 10-fold cross validation runs on the DLBCL training data. Preliminary analyses showed a *cutPoint *of 60 yielded better results than either 40 or 50 (data not shown), and furthermore, a threshold of 60% has precedence in the literature (e.g., [33]). As noted previously, the leaps and bounds algorithm from Furnival and Wilson [55] becomes inefficient for BMA windows larger than 30 variables. On training sets with relatively small numbers of samples (e.g., the breast cancer dataset used in this work), *maxNvar *may need to be reduced below the 30-variable limit in order to avoid convergence errors caused by matrix singularity and instability in fitting the data. For this reason, we have chosen a conservative default value of 25 for *maxNvar*. A window size of 25 provides a good balance between approximating the maximum and avoiding convergence errors. Our initial cross validation runs also showed that *p *< 500 performed poorly, while *p *> 1000 did not add significant predictive value beyond that of the first 1000 genes. Table 2 presents the results from 10 runs of 10-fold cross validation with *nbest *= 10, 20, 50, and 100 for both *p *= 500 and *p *= 1000 genes on the DLBCL dataset. The means and standard deviations of the p-values and chi-square statistics are calculated across all folds and all runs for each line in the table. As shown in Table 2, the parameters p = 1000 and nbest = 50 produced the lowest average p-value.

**Table 2:** 10-run/10-fold cross validation results on the DLBCL dataset for *cutPoint *= 60 and *maxNvar *= 25.

*p* (# genes)	*nbest*	Average p-value	p-value stdev	Average chi-square value	chi-square stdev
500	10	0.385	0.308	2.048	2.831
500	20	0.398	0.313	1.853	2.349
500	50	0.329	0.291	2.211	2.842
500	100	0.320	0.294	2.414	2.735

1000	10	0.313	0.303	2.648	3.139
1000	20	0.369	0.308	2.107	2.588
1000	50	0.307	0.303	2.958	3.251
1000	100	0.310	0.271	2.493	3.040

For the results shown in the rest of this paper, we adopted the optimal input parameters (*p *= 1000 and *nbest *= 50) determined from the 10-run/10-fold cross validation study on the DLBCL dataset shown in Table 2 in addition to the chosen 60% *cutPoint *to define risk groups. On the DLBCL data, we used the default BMA window size (represented by the input parameter *maxNvar*) of 25. However, on the breast cancer data, we were unable to use the default *maxNvar *value of 25. During preliminary analyses (data not shown), we found that training sets with fewer than 100 samples tended to result in fatal errors at higher values of *maxNvar*. These errors occur because smaller matrices lead to instability in fitting the data. These singularity errors can be largely mitigated by reducing the number of variables in the active BMA window. Since the breast cancer training set is relatively small (61 samples vs. 160 samples in the DLBCL data), we reduced the value of *maxNvar *from 25 to 15 variables in order to avoid convergence errors caused by matrix singularity.

## Results and discussion

### Breast Cancer Data

We applied *iterativeBMAsurv *to the breast cancer dataset of van't Veer et al. [27] using parameters p = 1000, nbest = 50, maxNvar = 15 and cutPoint = 60, and the algorithm selected a total of 15 genes across 84 contending models. Please refer to the Methods section for a detailed discussion of the selection of input parameters. The number of variables per model ranged from 5 to 10, with an average of 8.37 genes per model. Table 3 shows the posterior probabilities, univariate log likelihood rankings, and descriptions of the 15 selected genes. This table shows that most of the selected genes have poor univariate rankings: the highest-ranked gene in this group is number 437 out of 1000, and genes ranked 993 through 1000 are all included in this set of selected predictive variables. Of the 15 genes selected by BMA, only 4 of them (27%) were assigned a univariate ranking above 900 by the Cox Proportional Hazards Model. Furthermore, the average ranking of the five genes with a posterior probability of 100.0% is 646.8. Since the iterative BMA algorithm ranks each individual gene in descending order of the log likelihood resulted from fitting the Cox proportional hazards model to each individual gene in the pre-processing step (see Figure 1), we compared the magnitudes of the log likelihood of the top ranked gene to the genes selected by BMA. Our detailed analysis showed that the univariate Cox proportional hazards model yielded similar log likelihoods across the top 1000 genes. Specifically, the log likelihoods for the top univariate ranked gene NM_012429, the 437-ranked gene NM_000767 (from Table 3), and the 1000-ranked gene NM_012415 (from Table 3) are -76.66, -86.53 and -88.40 respectively. Hence, on this dataset, the poorly-ranked univariate genes produce comparable goodness-of-fit to the top ranked genes. As a result, it is not surprising that the BMA selected genes with poor univariate rankings achieve substantial predictive power when considered in combinations.

**Table 3:** Genes selected by the iterative BMA algorithm and their corresponding posterior probabilities, univariate log likelihood rankings, and descriptions on the breast cancer data (*p *= 1000, *nbest *= 50, *maxNvar *= 15, and *cutPoint *= 60).

Selected genes	Posterior Probability (%)	Univariate Cox ranking	Gene description
NM_000767	100.0	437	cytochrome P450, subfamily IIB (phenobarbital-inducible)
NM_002019	100.0	533	fms-related tyrosine kinase 1 (vascular endothelial growth factor/vascular permeability factor receptor)
Contig47102_RC	100.0	564	no description available
NM_013989	100.0	765	deiodinase, iodothyronine, type II (DIO2), transcript variant 1, mRNA
NM_018965	100.0	935	triggering receptor expressed on myeloid cells 2
NM_021151	99.0	956	carnitine O- octanoyltransferase
AF063936	43.9	984	putative neuronal cell adhesion molecule
NM_004911	40.6	998	protein disulfide isomerase related protein (calcium-binding protein, intestinal-related)
NM_014862	29.5	994	KIAA0307 gene product
Contig40146	19.0	996	wi84e 12.x1 NCI_CGAP_Kid12 Homo sapiens cDNA clone IMAGE : 2400046 3' similar to SW: RASD_DICDI P03967 RAS- LIKE PROTEIN RASD;, mRNA sequence
NM_012319	17.6	993	LIV-1 protein, estrogen regulated
NM_002411	13.1	995	secretoglobin, family 2A, member 2 (SCGB2A2), mRNA
NM_003645	10.7	997	fatty-acid-Coenzyme A ligase, very long-chain 1
NM_012415	10.3	1000	RAD54 homolog B (S. cerevisiae), transcript variant 1, mRNA (cDNA Clone, ORF Clone)
NM_015972	9.4	999	polymerase (RNA) I polypeptide D, 16 kDa

The genes are sorted first in descending order of their posterior probabilities and second in ascending order of their univariate rankings.

The maximum likelihood estimate coefficients of the 15 selected genes and the posterior probabilities of the 84 selected models were used to compute the predicted risk scores of the 234 patients in the validation dataset. We computed the 60% cutoff point using the risk scores of the patients in the training set, and the test samples with predicted risk scores under the cutoff point were placed in the low-risk group. The patients whose risk scores exceeded the *cutPoint *were designated as high-risk. Since the overall prognosis for breast cancer is fairly promising relative to other types of cancer, 179 patients (76.5%) were still alive at the conclusion of the study. The iterative BMA algorithm assigned 127 patients to the low-risk category and 107 to the high-risk category. Of the 179 patients that survived, 110 (61%) were placed in the low-risk group, while 69% (38/55) of the patients that succumbed to their disease were high-risk members. Table 4 shows the number of patients in each group: high-risk/censored, high-risk/uncensored, low-risk/censored, and low-risk/uncensored, in addition to the corresponding margin totals.

**Table 4:** The number of censored and uncensored breast cancer patient samples in each risk group, along with the total number of censored and uncensored patients and the total number of patients in the high- and low-risk categories.

	Censored	Uncensored	Total
High risk	69	38	107
Low risk	110	17	127
Total	179	55	

**Figure 2:**
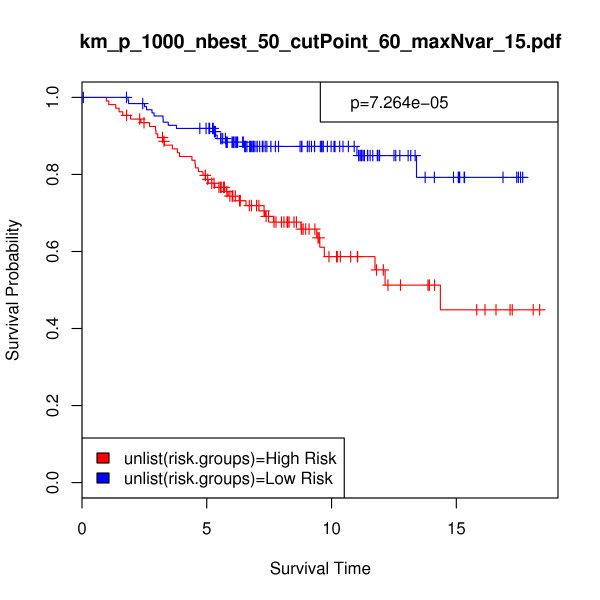
**Breast cancer data, n = 234: Kaplan-Meier survival analysis curve as a nonparametric estimator of the difference between risk groups**. In this analysis, *p *= 1000, *nbest *= 50, *maxNvar *= 15, and *cutPoint *= 60. Validation set risk scores were predicted using 15 selected genes across 84 selected models. Survival time is given in years, p-value = 7.26e-05, and chi-square = 15.741.

Figure 2 shows the Kaplan-Meier survival analysis curve in which the proportion of surviving patients from each risk group is plotted against time. For the breast cancer validation samples, we found a p-value of 7.264e-05 and a chi-square statistic of 15.741 from the log-rank test. Figure 2 and the highly significant associated p-value (< 0.0001) show that our method assigned the validation patient samples to relatively distinct risk groups. Furthermore, our results compared favorably with previous work. For example, Bair and Tibshirani [35] applied their semi-supervised principal components method to the full dataset of van de Vijver et al. [49]. Recall that this dataset is comprised of 78 training samples and 295 test samples, 61 of which overlap. When testing the difference between risk groups on the full validation set of 295 patients, Bair & Tibshirani used only five predictor genes and reported a p-value of 3.12e-05 from the log-rank test. They subsequently removed the 61 overlapping samples and applied their method to the validation set of 234 independent samples used in this work. With the exclusion of the overlapping records, their p-value increased to 0.00328. For comparison purposes, Bair & Tibshirani also analyzed the difference between risk groups for both n = 234 and n = 295 as calculated through the discrete predictor method described in van't Veer et al. [27]. They reported p-values of 0.0105 and 0.00934 respectively.

**Figure 3:**
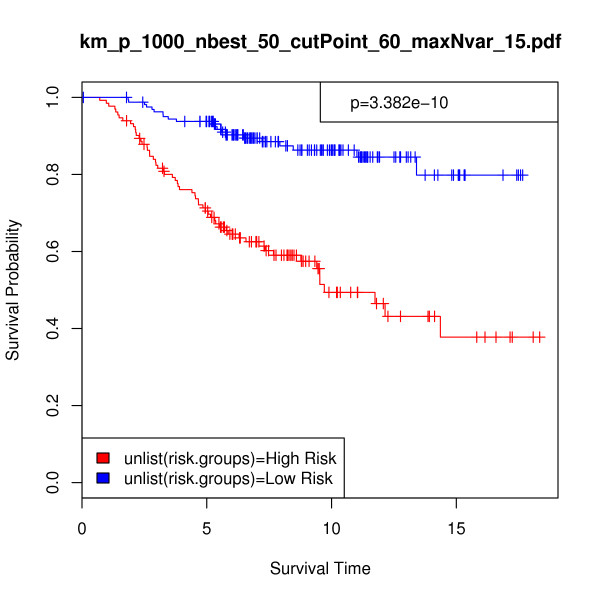
**Breast cancer data, n = 295: Kaplan-Meier survival analysis curve calculated on the full 295-sample breast cancer validation set of van de Vijver et al**. [49]. In this analysis, *p *= 1000, *nbest *= 50, *maxNvar *= 15, and *cutPoint *= 60. Validation set risk scores were predicted using 15 selected genes across 84 selected models. Survival time is given in years; p-value = 3.38e-10 and chi-square = 39.441.

**Figure 4:**
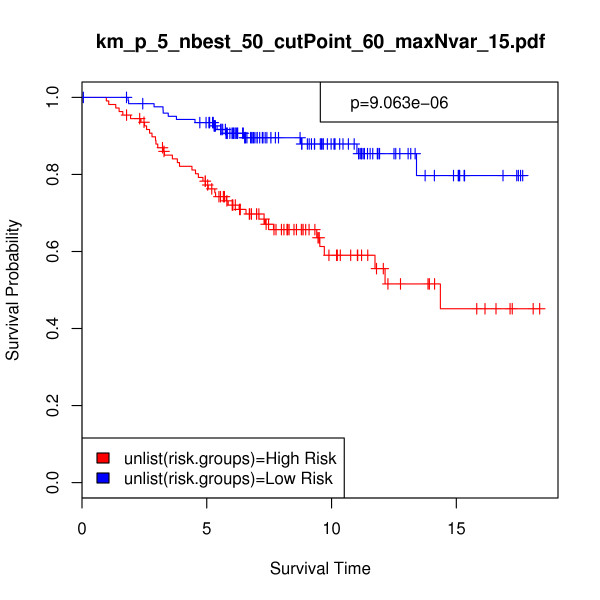
**5-gene Breast cancer data, n = 234: Kaplan-Meier survival analysis curve as a nonparametric estimator of the difference between risk groups**. In this analysis, *p *= 5, *nbest *= 50, *maxNvar *= 15, and *cutPoint *= 60. Validation set risk scores were predicted using 5 top-ranked genes across 2 selected models. Survival time is given in years, p-value = 9.06e-06, and chi-square = 19.699.

**Figure 5:**
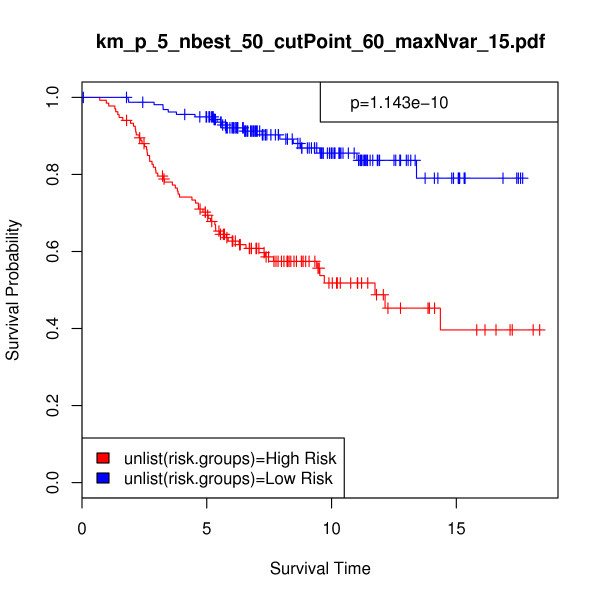
**5-gene Breast cancer data, n = 295: Kaplan-Meier survival analysis curve calculated on the full 295-sample breast cancer validation set of van de Vijver et al**. [49]. In this analysis, *p *= 5, *nbest *= 50, *maxNvar *= 15, and *cutPoint *= 60. Validation set risk scores were predicted using 5 top-ranked genes across 2 selected models. Survival time is given in years, p-value = 1.14e-10, and chi-square = 41.559.

In order to provide a more direct performance comparison between the iterative BMA method and these alternative procedures, we made some modifications. First, we applied the previously selected 15 genes and 84 models to the full van de Vijver et al. validation set of 295 samples. Figure 3 displays the resulting Kaplan-Meier survival analysis curve (p-value = 3.382e-10, chi-square = 39.441). Second, we predicted the risk scores for the validation set and calculated the difference between the risk groups using the top 5 genes with posterior probabilities of 100% from Table 3. Figure 4 shows the Kaplan-Meier survival analysis curve using these 5 genes for n = 234 (p-value = 9.063e-06, chi-square = 19.699), and Figure 5 provides the same information for n = 295 (p-value = 1.143e-10, chi-square = 41.559). The exclusion of the bottom-ranked 10 genes did not undermine predictive accuracy; in fact, the results are slightly better than those obtained from using all 15 genes originally selected by the algorithm.

Furthermore, we investigated the effect of the adaptive threshold heuristic in which the posterior probability threshold is increased temporarily to ensure that at least one gene is removed in each iteration. Our exploration showed that the adaptive threshold played an important role in the results shown in Table 3. Specifically, many of the high univariate ranked genes were removed due to the adaptive threshold. If we turned off the adaptive threshold heuristic, there will only be a single iteration of bic.surv since all the top univariate ranked genes produced posterior probabilities greater than the 1% threshold. Without the adaptive threshold, our analysis showed that using the top 15 univariate genes produced less distinct risk groups (p-value = 6.21e-4, chi-square = 11.712 for n = 234). We have also explored the keepRmModels=TRUE heuristic in which all the models discarded due to the adaptive threshold were re-considered by the Occam's window method. In this case, our analysis yielded mostly high-ranked univariate genes (32 genes spanning across 217 models) that produced slightly less distinct risk groups (p-value = 2.31e-3, chi-square = 9.283 for n = 234). Please refer to Additional file 1 for more detailed results using this heuristic.

Table 5 summarizes the comparison in terms of the p-values, chi-square statistics and numbers of predictor genes across all aforementioned methodologies for the two different breast cancer validation sets. As shown in Table (5), our iterative BMA algorithm produced lower p-values and higher chi-square statistics than the other two studies using only 5 predictor genes.

**Table 5:** A comparison of the p-values and chi-square statistics from the log-rank test and numbers of genes selected across different survival analysis methods on the full breast cancer validation set of van de Vijver et al. [49] (n = 295), and the partial breast cancer validation set used in this work with 61 overlapping samples removed (n = 234).

	**heuristic**	**# genes**	**n = 234**	**n = 234**	**n = 295**	**n = 295**
			**p-value**	**chi-square**	**p-value**	**chi-square**
**iterative BMA**	adaptive threshold, keepRmModels = FALSE	15	7.264E-05	15.714	3.382E-10	39.441
**iterative BMA**	top 5 genes with 100% posterior probabilities	5	**9.063E-06**	**19.699**	**1.143E-10**	**41.559**
**iterative BMA**	adaptive threshold, keepRmModels = TRUE	32	2.312E-03	9.283	9.875E-08	28.398
**Bair & Tibshirani (2002) Principle Components**	NA	5	3.280E-03	8.645*	3.120E-05	17.343*
**Method of van't Veer et al. (2002) (as calculated by Bair & Tibshirani)**	NA	70	1.050E-02	6.548*	9.340E-03	6.757*

The chi-square statistics marked with an asterisk (*) are computed using the p-values since the chi-square statistics are not directly available from Bair and Tibshirani [35]. Our iterative BMA method using the adaptive threshold produced the lowest p-value and highest chi-square statistic (shown in bold) using the smallest number of predictor genes.

### DLBCL Data

For the DLBCL data, the iterative BMA algorithm for survival analysis with the adaptive threshold heuristic using *p *= 1000, *nbest *= 50, *maxNvar *= 25 and *cutPoint *= 60 selected a total of 25 predictive genes contained within 3 contending models. The models contained 24, 23, and 25 genes respectively. Of the 25 genes selected, 23 had a posterior probability of 100.0%, which means that these 23 genes were included in all three models. Table 6 lists these 25 selected genes, along with their descriptions, posterior probabilities, and univariate log likelihood rankings. This table also demonstrates that genes with poor univariate rankings may be selected by our BMA algorithm. For example, both the highest-ranked gene (BC012161) and the lowest-ranked gene (U70981) in the top *p *= 1000 genes were included in the final set of predictive variables. In addition, several genes with rankings between 500 and 1000 were returned with calculated posterior probabilities of 100.0% (e.g., D83492, AK025754, and NM_005347).

**Table 6:** Genes selected by the iterative BMA algorithm and their corresponding posterior probabilities, univariate log likelihood rankings, and descriptions on the DLBCL dataset (*p *= 1000, *nbest *= 50, *maxNvar *= 25, and *cutPoint *= 60).

Selected genes	Posterior Probability (%)	Univariate Cox ranking	Gene description
BC012161	100.0	1	septin 1
D42043	100.0	4	KIAA0084 protein
X53505	100.0	41	ribosomal protein S12
BF129543	100.0	49	ESTs, weakly similar to A47224 thyroxine-binding globulin precursor
D13666	100.0	73	osteoblast specific factor 2 (fasciclin I-like)
M83664	100.0	93	MHC, class II, DP beta I
AK000978	100.0	101	hypothetical protein FLJ10116
AF009615	100.0	116	a disintegrin and metallo- proteinase domain 10
AK027711	100.0	123	hypothetical protein MGC3234
LC_24015	100.0	129	no description available
K01144	100.0	140	CD74 antigen (invariant polypeptide of MHC, class II antigen associated)
U68418	100.0	181	branched chain aminotransferase 2
D88532	100.0	213	phosphoinositide-3-kinase, regulatory subunit, polypeptide 3 (p55, gamma)
NM_022551	100.0	223	ribosomal protein S18
U18259	100.0	242	MHC, class II transactivator
X64707	100.0	243	ribosomal protein L13
NM_006312	100.0	278	nuclear receptor co- repressor 2
M58297	100.0	385	zinc finger protein 42 (myeloid- specific retinoic acid-responsive)
LC_26524	100.0	473	no description available
AK022743	100.0	499	hypothetical protein FLJ12681
NM_005347	100.0	518	heat shock 70 kDa protein 5 (glucose-regulated protein 78 kDa)
D83492	100.0	632	EphB
AK025754	100.0	652	HP1-BP74
AA747694	81.7	885	ESTs, weakly similar to ALU SUBF J
U70981	9.2	1000	interleukin 13 receptor, alpha 2

The genes are sorted in descending order of their posterior probabilities and ascending order of their univariate rankings.

Our algorithm computed the predicted risk scores of the test samples using the maximum likelihood estimate coefficients of the selected genes and the posterior probabilities of the selected models. The risk groups were assigned using the 60% cutoff point of the calculated risk scores in the training set. Of the 80 samples in the validation dataset, 24 were assigned to the high-risk category while 56 were deemed low-risk. Only 3 patients in the high-risk group were still alive at the final follow-up visit, while 27 low-risk patients survived to the study's conclusion. Table 7 provides a summary of the patient samples in each category, along with the margin totals. The majority of patients in the DLBCL validation dataset did not survive (50/80 = 62.5%), which explains the relatively large number of dead patient samples assigned to the low-risk group. Of the 30 patients who were alive at the final visit, only 3 (10%) were placed in the high-risk category.

**Table 7:** The number of censored and dead DLBCL patients in each risk group, along with the total number of censored and dead patients and the total number of patients in the high- and low-risk categories.

	Censored	Died	Total
High risk	3	21	24
Low risk	27	29	56
Total	30	50	

**Figure 6:**
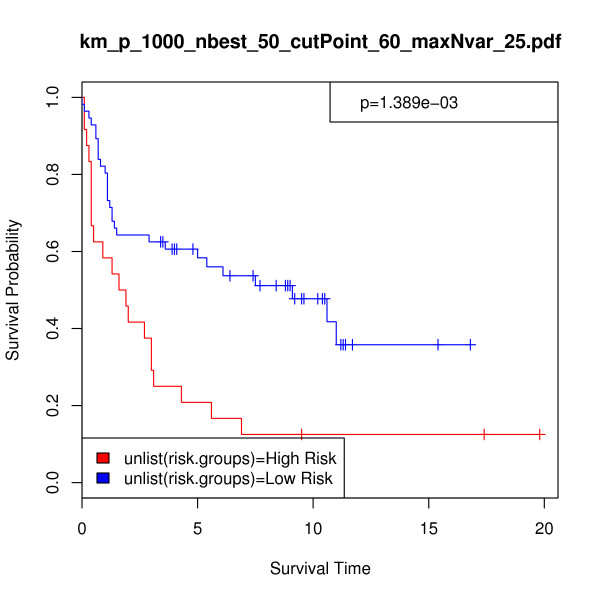
**DLBCL data: Kaplan-Meier survival analysis curve as a nonparametric estimator of the difference between risk groups**. In this analysis, *p *= 1000, *nbest *= 50, *maxNvar *= 25, and *cutPoint *= 60. Survival time is given in years, p-value = 0.00139, and chi-square = 10.221.

To assess the difference between high- and low-risk patient categories, the p-value from the log-rank test was calculated using the central chi-square distribution. The two risk groups showed a significant difference in survival probability at p-value = 0.00139 and chi-square = 10.221. Figure 6 shows the Kaplan-Meier survival analysis curve, where survival time is given in years. These results are comparable with previous studies using the same division between the training and testing sets on the DLBCL data. Rosenwald et al. [50] identified four separate gene-expression signatures within the patient samples, and the number of microarray features within each signature ranged from 37 to 1333. The p-values illustrating the difference between high-, medium-, and low-risk validation samples in each of the four signatures ranged from 0.009 to 0.11. Bair and Tibshirani [35] used a semi-supervised principal components method to separate the validation set of 80 DLBCL patients into high- and low-risk groups. They used 17 genes in their analysis and reported a p-value of 0.00124. These results are summarized in Table 8.

**Table 8:** A comparison among three studies of the number of genes selected, the corresponding p-values and chi-square statistics in survival analysis on the DLBCL dataset.

	**Number of Genes**	**p-value**	**chi-square**
**iterativeBMA**	25	0.00139	10.221
**Bair & Tibshirani (2004)**	17	0.00124	10.430*
**Rosenwald et al. (2002)**	37–1333	0.009 – 0.11	2.554 – 6.823*

The chi-square statistics marked with an asterisk (*) are computed using the p-values since the chi-square statistics are not directly available from Bair and Tibshirani [35].

As with the breast cancer data, we restricted the number of predictor variables in the DLBCL training set to include only those genes with posterior probabilities of 100%. Because 23 of the 25 genes originally selected by *iterativeBMAsurv *belonged in this category, the outcome was almost identical to the results presented in Figure 6. This information suggests that re-running the algorithm with a reduced set of predictor variables may be more worthwhile when relatively fewer genes are calculated to have the maximum posterior probability.

### Comparison with Other Methods

Here we compared our iterativeBMAsurv results to that from using ridge regression which was shown to produce the best overall prediction accuracy in a recent empirical study from Bovelstad *et al*. [42]. Specifically, Bovelstad *et al*. compared the prediction performance of seven methods that are based on the Cox proportional hazards model, including univariate selection, forward selection, principal components regression, supervised principal components regression, partial least squares regression, ridge regression and LASSO. Since Bovelstad et al. reported results produced by randomly splitting three microarray datasets into training and test sets, our results are not directly comparable with theirs. Therefore, we downloaded their software implementation written in Matlab from their supplementary web site [64], ran their software on the breast cancer and DLBCL datasets from which we derived our results, and then assessed the results using the log-rank test. On the breast cancer data, ridge regression produced a p-value of 0.00340 using all 4919 genes. In contrast, our iterativeBMAsurv algorithm produced a much more significant p-value of 9.063e-06 using only 5 genes. On the DLBCL data, ridge regression produced a p-value of 0.000380 using all 7399 genes. In contrast, our iterativeBMAsurv algorithm produced a p-value of 0.001389 using 25 genes. Bovelstad *et al*. focused on prediction accuracy instead of the number of selected genes. In summary, ridge regression may produce good prediction accuracy, but it is not a variable selection algorithm. On the other hand, our iterativeBMAsurv algorithm typically selects a small number of genes and produces good prediction accuracy.

## Conclusion

In this paper, we have proposed an extension of the iterative BMA algorithm of Yeung et al. [19] for application to survival analysis with high-dimensional microarray data. This multivariate technique accounts for model uncertainty by averaging over the posterior probabilities of the strongest contending models (sets of potentially overlapping predictor genes). We have demonstrated that our *iterativeBMAsurv *algorithm achieves highly significant p-values and larger chi-square statistics from the log-rank test on the breast cancer data when compared to alternative methods using the same number of predictor genes. In addition, our algorithm produces similar results on the DLBCL dataset when compared to the best method in the literature. Table 9 provides a summary of our results. The output of the iterative BMA algorithm for survival analysis is particularly well suited to biological interpretation. The posterior probability of a chosen gene represents its overall contribution towards the patient risk score across all selected models. The posterior probabilities of the chosen models indicate the relative strength of the predictor genes from each model in patient risk assessment. The models chosen for the breast cancer and DLBCL datasets are relatively simple, consisting of anywhere from 5 to 25 genes. The p-values and Kaplan-Meier survival analysis curves are used to estimate the difference between risk groups, demonstrating the strength of the iterative BMA algorithm as measures of high predictive accuracy.

**Table 9:** A summary of the results from the application of the iterative BMA algorithm to the DLBCL dataset, the partial non-overlapping breast cancer dataset (n = 234), and the full overlapping breast cancer dataset (n = 295).

	Number of Genes	Number of Models	p-value	chi-square
DLBCL	25	3	1.389e-03	10.221
				
Breast Cancer n = 234 (15 genes)	15	84	7.264e-05	15.714
				
Breast Cancer n = 295 (15 genes)	15	84	3.382e-10	39.441
				
Breast Cancer n = 234 (5 genes)	5	2	9.063e-06	19.699
				
Breast Cancer n = 295 (5 genes)	5	2	1.143e-10	41.559

Our results showed that genes with poor univariate rankings are often selected by the iterative BMA algorithm using the adaptive threshold heuristic (see Tables 3 and 6). Our analysis showed that genes with dramatic difference in the univariate rankings may in fact have similar goodness-of-fit (i.e. comparable log likelihood) when fitted to the univariate Cox proportional hazards model. On the breast cancer data, we showed that our selected set of genes (with poor univariate rankings) resulted in patients being assigned to more distinct risk groups than the top univariate genes. While it is true that the genes and models selected by the iterative BMA procedure are contingent upon the initial univariate rankings, all *p *top-ranked univariate genes may be included in the models selected by our *iterativeBMAsurv *method. Our results showed that setting the parameter *p *to a large value (e.g., 1000) generally yields high prediction accuracy. In addition to the parameter *p*, our algorithm requires the input of a few other user-specified parameters. One example is *nbest*, which is used by the leaps and bounds algorithm from Furnival and Wilson [55] to isolate the *nbest *strongest models. Higher values of *nbest *increase the computation time, but overly restrictive values undermine predictive accuracy by failing to return potentially contributory models. We found that *nbest *= 20, 50, and 100 generally yielded good results, with a value of 50 exhibiting the ideal tradeoff between predictive power and computational efficiency on the DLBCL data. For example, it takes about 1.5 hours to run the iterative BMA algorithm with *p *= 1000 genes and *nbest *= 50 on a machine with 2 gigabytes of RAM and a 2.0 GHz Intel dual core processor. Reducing *nbest *to 20 cuts the running time down to 40 minutes, but the p-value and chi-square statistic representing the difference between risk groups is slightly less favorable. On the other hand, setting *nbest *to 100 significantly increases the computation time with no appreciable improvement in prediction. Cross validation can be used to determine the optimal input parameters for each dataset.

While the results obtained in this study are encouraging, the iterative BMA algorithm for survival analysis presents some limitations and areas for future development. The mathematical calculations conducted by the BMA methods are close approximations, but they could be computed with greater precision. For example, the maximum likelihood estimate of equation (3) provides a sufficient approximation to the predictive distribution, but the more computationally intensive Markov Chain Monte Carlo methods might yield the true predictive distribution with greater accuracy [31]. The approximation of the posterior model probabilities calculated in equation (6) could also be improved [62]. Another area for future work lies in the assessment of the performance from different computational methods. Currently, computational methods are evaluated by comparing the separation between different risk groups using the log-rank test (e.g. [35,50]). In our work, we divided patients into the high and low risk groups using cutPoint = 60% and evaluated computational methods based on the p-values and chi-square statistics computed using the log-rank test. Note that we are only comparing the p-values over identical test sets. Comparing p-values across different test sets could potentially be mis-leading, and hence, we recommend using both the p-values and chi-square statistics when evaluating different computational methods. Further work is required to investigate the optimal number of risk groups and to propose statistical methods for the assessment of different computational methods across different test sets.

The iterative BMA algorithm for survival analysis is easy to use, computationally efficient, and highly accurate. It identifies a handful of predictor variables from vast amounts of microarray data, making it a cost-effective diagnostic tool in the clinical setting. In terms of future work, we would like to collaborate with cancer biologists to validate the predictor genes selected by applying the *iterativeBMAsurv *algorithm to microarray data, and to assess the prediction accuracy of our methodology on PCR data generated using independent patient samples. Furthermore, we would like to extend the iterative BMA algorithm to other types of high-throughput data such as proteomics data produced from mass spectrometry. The multivariate nature of BMA combined with its ability to account for model uncertainty makes it an attractive candidate to extract predictive genes from any high-dimensional biological data.

All analyses in this study were conducted using R statistical software . The Bioconductor packages for the iterative BMA algorithms for classification and survival analysis described in this paper are available for download from Bioconductor's website  as the *iterativeBMA *and *iterativeBMAsurv *packages respectively. Please visit our supplemental website for access to the breast cancer and DLBCL datasets, along with other helpful links and information.

## Supplemental website

**URL: **

## Software

Our software implementation is publicly available as a bioconductor package called "iterativeBMAsurv" 

## Abbreviations

AFT: accelerated failure time; BMA: Bayesian Model Averaging; BSS/WSS: between-groups to within-groups sum of squares ratio; DLBCL: diffuse large B-cell lymphoma; iterativeBMAsurv: iterative Bayesian Model Averaging for survival analysis; LASSO: least absolute shrinkage and selection operator; PAM: Prediction Analysis for Microarrays; PLS: partial least squares; PPS: partial predictive score; RFE: recursive feature elimination; SAM: Significance Analysis for Microarrays; TNoM: threshold number of misclassification score

## Authors' contributions

AA carried out the software implementation, data analysis, drafted the manuscript and set up the supplementary web site. REB and AER provided guidance for the study, and edited the manuscript. AER conceived the project and developed the original BMA algorithm. KYY coordinated the project and software implementation, edited and prepared the manuscript for submission. All authors read and approved the final manuscript.

## Supplementary Material

Additional file 1 **Supplementary Materials**. This document contains supplementary tables, figures and method. Click here for file
