# Supplementary material for: Iterative Bayesian Model Averaging: a method for the application of survival analysis to high-dimensional microarray data
Source: BMC Bioinformatics. 2009 Feb 26;10:72. doi: 10.1186/1471-2105-10-72 (PMC2657791; doi:10.1186/1471-2105-10-72)

Supplementary Materials

Iterative Bayesian Model Averaging: A Method for the Application of Survival Analysis to High-Dimensional Microarray Data

Amalia Annest, Roger E. Bumgarner, Adrian E. Raftery, and Ka Yee Yeung

This document contains supplementary tables, figures and method

SUPPLEMENTARY TABLES

Table S1. 10-run/10-fold cross validation results on the DLBCL dataset for *cutPoint*=60 and *maxNvar*=25.

p (# genes)	*nbest*	Average p-value	p-value stdev	Average chi-square value	chi-square stdev
500	10	0.385	0.308	2.048	2.831
500	20	0.398	0.313	1.853	2.349
500	50	0.329	0.291	2.211	2.842
500	100	0.320	0.294	2.414	2.735
1000	10	0.313	0.303	2.648	3.139
1000	20	0.369	0.308	2.107	2.588
1000	50	0.307	0.303	2.958	3.251
1000	100	0.310	0.271	2.493	3.040

Table S2. Genes selected by the iterative BMA algorithm and their corresponding posterior probabilities, univariate log likelihood rankings, and descriptions. Analysis was conducted on the breast cancer dataset with *p*=1000, *nbest*=50, *maxNvar*=15, and *cutPoint*=60. The genes are sorted first in descending order of their posterior probabilities and second in ascending order of their univariate rankings.

Selected genes	Posterior Probability (%)	Univariate Cox ranking	Gene description
NM_000767	100.0	437	cytochrome P450, subfamily IIB (phenobarbital-inducible)
NM_002019	100.0	533	fms-related tyrosine kinase 1 (vascular endothelial growth factor/vascular permeability factor receptor)
Contig47102_RC	100.0	564	no description available
NM_013989	100.0	765	deiodinase, iodothyronine, type II (DIO2), transcript variant 1, mRNA
NM_018965	100.0	935	triggering receptor expressed on myeloid cells 2
NM_021151	99.0	956	carnitine O-octanoyltransferase
AF063936	43.9	984	putative neuronal cell adhesion molecule
NM_004911	40.6	998	protein disulfide isomerase related protein (calcium-binding protein, intestinal-related)
NM_014862	29.5	994	KIAA0307 gene product
Contig40146	19.0	996	wi84e 12.x1 NCI_CGAP_Kid12 Homo sapiens cDNA clone IMAGE : 2400046 3' similar to SW: RASD_DICDI P03967 RAS-LIKE PROTEIN RASD;, mRNA sequence
NM_012319	17.6	993	LIV-1 protein, estrogen regulated
NM_002411	13.1	995	secretoglobin, family 2A, member 2 (SCGB2A2), mRNA
NM_003645	10.7	997	fatty-acid-Coenzyme A ligase, very long-chain 1
NM_012415	10.3	1000	RAD54 homolog B (S. cerevisiae), transcript variant 1, mRNA (cDNA Clone, ORF Clone)
NM_015972	9.4	999	polymerase (RNA) I polypeptide D, 16kDa

Table S3. The number of censored and uncensored breast cancer patient samples in each risk group, along with the total number of censored and uncensored patients and the total number of patients in the high- and low-risk categories.

	Censored	Uncensored	Total
High risk	69	38	107
Low risk	110	17	127
Total	179	55	

Table S4. Genes selected by the iterative BMA algorithm and their corresponding posterior probabilities, univariate log likelihood rankings, and descriptions. The analysis was conducted on the DLBCL dataset with *p*=1000, *nbest*=50, *maxNvar*=25, and *cutPoint*=60. The genes are sorted in descending order of their posterior probabilities and ascending order of their univariate rankings.

Selected genes	Posterior Probability (%)	Univariate Cox ranking	Gene description
BC012161	100.0	1	septin 1
D42043	100.0	4	KIAA0084 protein
X53505	100.0	41	ribosomal protein S12
BF129543	100.0	49	ESTs, weakly similar to A47224 thyroxine-binding globulin precursor
D13666	100.0	73	osteoblast specific factor 2 (fasciclin I-like)
M83664	100.0	93	MHC, class II, DP beta I
AK000978	100.0	101	hypothetical protein FLJ10116
AF009615	100.0	116	a disintegrin and metallo-proteinase domain 10
AK027711	100.0	123	hypothetical protein MGC3234
LC_24015	100.0	129	no description available
K01144	100.0	140	CD74 antigen (invariant polypeptide of MHC, class II antigen associated)
U68418	100.0	181	branched chain aminotransferase 2
D88532	100.0	213	phosphoinositide-3-kinase, regulatory subunit, polypeptide 3 (p55, gamma)
NM_022551	100.0	223	ribosomal protein S18
U18259	100.0	242	MHC, class II transactivator
X64707	100.0	243	ribosomal protein L13
NM_006312	100.0	278	nuclear receptor co-repressor 2
M58297	100.0	385	zinc finger protein 42 (myeloid-specific retinoic acid-responsive)
LC_26524	100.0	473	no description available
AK022743	100.0	499	hypothetical protein FLJ12681
NM_005347	100.0	518	heat shock 70kDa protein 5 (glucose-regulated protein 78kDa)
D83492	100.0	632	EphB
AK025754	100.0	652	HP1-BP74
AA747694	81.7	885	ESTs, weakly similar to ALU SUBF J
U70981	9.2	1000	interleukin 13 receptor, alpha 2

Table S5. The number of censored and dead DLBCL patients in each risk group, along with the total number of censored and dead patients and the total number of patients in the high- and low-risk categories.

	Censored	Died	Total
High risk	3	21	24
Low risk	27	29	56
Total	30	50	

Table S6. A summary of the results from the application of the iterative BMA algorithm to the DLBCL dataset, the partial non-overlapping breast cancer dataset (n=234), and the full overlapping breast cancer dataset (n=295).

	Number of Genes	Number of Models	p-value	chi-square
DLBCL	25	3	1.389e-03	10.221
Breast Cancer n=234 (15 genes)	15	84	7.264e-05	15.714
Breast Cancer n=295 (15 genes)	15	84	3.382e-10	39.441
Breast Cancer n=234 (5 genes)	5	2	9.063e-06	19.699
Breast Cancer n=295 (5 genes)	5	2	1.143e-10	41.559

SUPPLEMENTARY FIGURES

Figure S1. Outline of the iterative BMA algorithm for survival analysis on microarray data.

Input: training set *TD* with *G* genes and *n* samples
Pre-processing step: Rank-order all *G* genes by applying Cox Proportional Hazards Regression to each individual gene. Let x_1, x_2, … , x_G be the ordered list of genes, sorted in descending order of log likelihood. Let *maxNvar* denote the user-specified size of the BMA window (maximum 30).
Parameters: *nbest* and *p*, where *p* is the total number of genes to be processed such that *maxNvar* < *p* ≤ *G*.

1. Initially, start with the *maxNvar* top ranked genes (x_1, x_2, …, $x_{maxNvar}$), and apply the traditional BMA algorithm for survival analysis (Volinsky et al., 1997). Let *toBeProcessed* be an ordered list of genes with ranks (*maxNvar* + 1) to *p*. Initially, *toBeProcessed* ← ($x_{maxNvar+1}$, x_{32}, …, x_p).
2. Repeat until all *p* genes are processed
    a. Remove all genes *i* with Pr(b_i ≠ 0 | *TD*) < 1%.
    b. *Adaptive threshold step*: If all genes have Pr(b_i ≠ 0 | *TD*) ≥ 1%, determine the minimum Pr(b_i ≠ 0 | *TD*), *minProbne0*, among the *maxNvar* genes in the current BMA window. Remove all genes with Pr(b_i ≠ 0 | *TD*) < (*minProbne0* + 1)%.
    c. Let *removedGenes* be the set of genes removed, and suppose *q* genes are removed.
    d. Replace the *q* removed genes with the *q-next-up* genes from *toBeProcessed*. Update *toBeProcessed* ← *toBeProcessed* – *q-next-up*.
    e. Apply the traditional BMA algorithm for survival analysis.

Output: selected models and their posterior probabilities, selected genes and their corresponding posterior probabilities (Pr(b_i ≠ 0 | *TD*)), maximum-likelihood estimates of the regression parameters in each model.

Figure S2. Breast cancer data, n=295: Kaplan-Meier survival analysis curve calculated on the full 295-sample breast cancer validation set of van de Vijver et al. [38]. In this analysis, *p*=1000, *nbest*=50, *maxNvar*=15, and *cutPoint*=60. Validation set risk scores were predicted using 15 selected genes across 84 selected models. Survival time is given in years; p-value=3.382e-10 and chi-square=39.441.

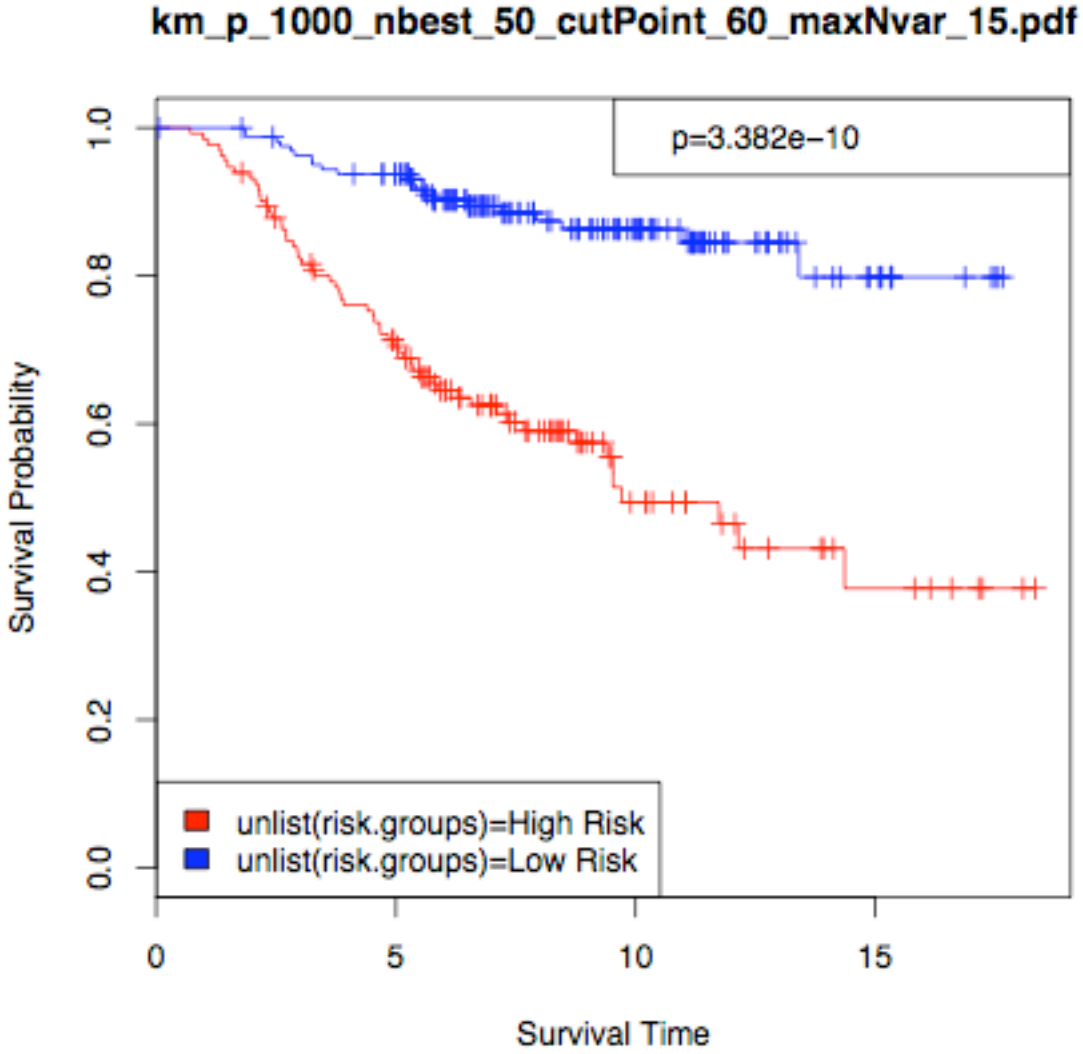

Figure S3. 5-gene Breast cancer data, n=295: Kaplan-Meier survival analysis curve calculated on the full 295-sample breast cancer validation set of van de Vijver et al. [38]. In this analysis, *p*=5, *nbest*=50, *maxNvar*=15, and *cutPoint*=60. Validation set risk scores were predicted using 5 top-ranked genes across 2 selected models. Survival time is given in years, p-value=1.143e-10, and chi-square=41.559.

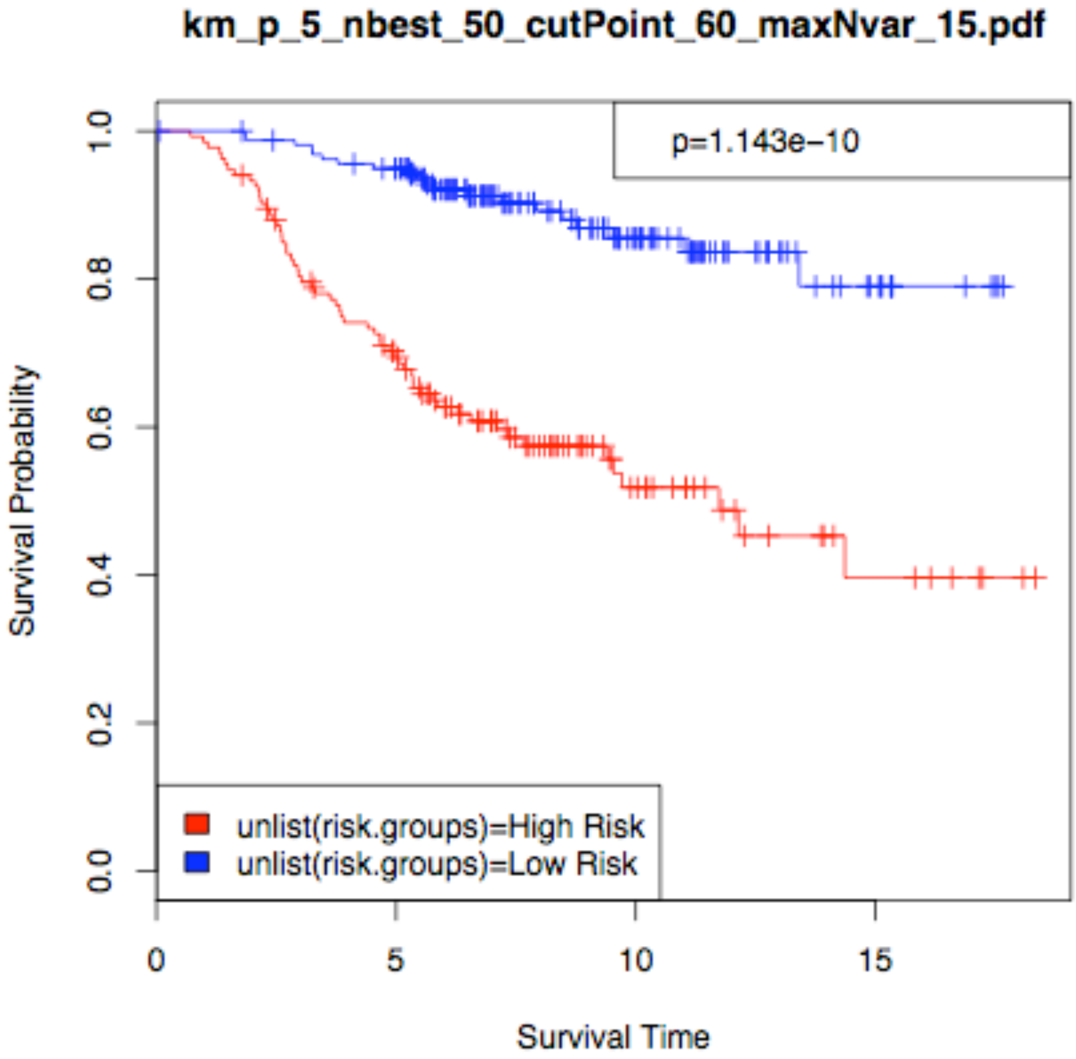

SUPPLEMENTARY METHOD

A. Selection of input parameters

The main user-specified parameters to the iterative BMA algorithm for survival analysis include the number of top-ranked *p* genes to be included in the iterations, the *nbest* strongest models to be returned by the leaps and bounds algorithm, the desired *cutPoint* for separating high- from low-risk patient samples, and the size of the active BMA window (*maxNvar*). In order to determine the best combination of these input parameters, we performed a series of 10-fold cross validation runs on the DLBCL training data. Preliminary analyses showed a *cutPoint* of 60 yielded better results than either 40 or 50 (data not shown). The leaps and bounds algorithm from Furnival and Wilson becomes inefficient for BMA windows larger than 30 variables. On training sets with relatively small numbers of samples (e.g., the breast cancer dataset used in this work), *maxNvar* may need to be reduced below the 30-variable limit in order to avoid convergence errors caused by matrix singularity and instability in fitting the data. For this reason, we have chosen a conservative default value of 25 for *maxNvar*. A window size of 25 provides a good balance between approximating the maximum and avoiding convergence errors. Our initial cross validation runs also showed that $p < 500$ performed poorly, while $p > 1000$ did not add significant predictive value beyond that of the first 1000 genes. Table S1 presents the results from 10 runs of 10-fold cross validation with *nbest*=10, 20, 50, and 100 for both *p*=500 and *p*=1000 genes on the DLBCL dataset. The means and standard deviations of the p-values and chi-square statistics are calculated across all folds and all runs for each line in the table. As shown in Table S1, the parameters p=1000 and nbest=50 produced the lowest average p-value.

B. keepRmModels=TRUE heuristic

Outline for keepRmModels=TRUE heuristic: Keeping track of the discarded models due to the adaptive threshold (set keepRmModels = TRUE)

1. Rank all p genes using univariate coxph
2. Repeat until all p genes are processed
    a. Apply bic.surv to the current window (maxNvar) genes
    b. Remove all genes with probne0 < 1%
    c. If there are no genes with probne0 < 1%, set adaptive threshold = 1 + min(probne0). Remove all genes with probne0 < adaptive threshold
    d. If the adaptive threshold kicks in, we want to keep track of all the models that got discarded. Use an extended "mle" matrix: (# models) * (p genes).
        i. Keep track of the estimated regression coeff from ret.bic.surv$mle
        ii. Also keep track of the BIC scores of the discarded models from ret.bic.surv$bic
3. Post-process the extended "mle" matrix
    a. Using Occam's windows: remove all models with BIC scores < (BIC_max–6)

b. compute the updated postprob for each remaining model from BIC. postprob = (exp(-(BIC)/2)) / (sum over all models exp(-(BIC)/2)) = (exp(-(BIC - BIC_max)/2)) / (sum over all models exp(-(BIC - BIC_max)/2))   (for numerical stability)
c. Remove all duplicated models
d. Compute the risk scores using the "mle" matrix and the computed postprob
e. Compute the updated probne0 for each gene (by summing up postprob from all models that the gene is involved in)

Summary of Results

	keepRmModels	# genes	# models	p-value	chi-square
**DLBCL**	no	25	3	1.39E-03	10.221
**Breast Cancer n=234**	no	15	84	7.26E-05	15.714
**Breast Cancer n=295**	no	15	84	3.38E-10	39.441
**Breast Cancer n=234 (5 genes)**	no	5	2	9.06E-06	19.699
**Breast Cancer n=295 (5 genes)**	no	5	2	1.14E-10	41.559
**DLBCL**	yes	38	55	4.45E-02	4.038
**Breast Cancer n=234**	yes	32	217	2.31E-03	9.283
**Breast Cancer n=295**	yes	32	217	9.88E-08	28.398
**Breast Cancer n=234 (5 genes)**	yes	5	7	9.35E-03	6.754
**Breast Cancer n=295 (5 genes)**	yes	5	7	6.83E-06	20.241

Breast cancer data n=234 with keepRmModels=T

selected genes = 32
selected models = 217
Risk Table:

	cens.vec.test 0	1
High Risk	67	33
Low Risk	112	22

chisq from the log-rank test = 9.28328108917349
pvalue from the log-rank test = 0.00231254825469163

gene name	posterior probability	univariate rank
AL133619	99.26%	8
NM_012429	93.36%	1
NM_001333	92.92%	15
Contig55377_RC	87.99%	10
NM_016267	79.04%	22

Contig36818_RC	77.42%	18
NM_001003	73.78%	28
U10991	70.66%	11
Contig49670_RC	69.71%	21
AB020689	53.75%	5
AL157492	51.62%	19
NM_000381	44.09%	2
NM_001673	21.04%	12
U82987	17.12%	31
AF111849	16.70%	17
Contig54394_RC	16.34%	29
NM_000600	11.68%	27
AL050372	11.67%	32
AF148505	9.01%	7
NM_000992	8.72%	26
X94232	8.60%	25
Contig60864_RC	4.50%	33
NM_016337	2.90%	13
NM_012067	2.30%	24
NM_004336	2.17%	23
Contig38288_RC	2.10%	20
AK000004	1.85%	16
NM_014246	1.47%	9
Contig55725_RC	1.46%	3
NM_000507	1.46%	6
NM_020974	1.45%	4
Contig58301_RC	1.33%	14

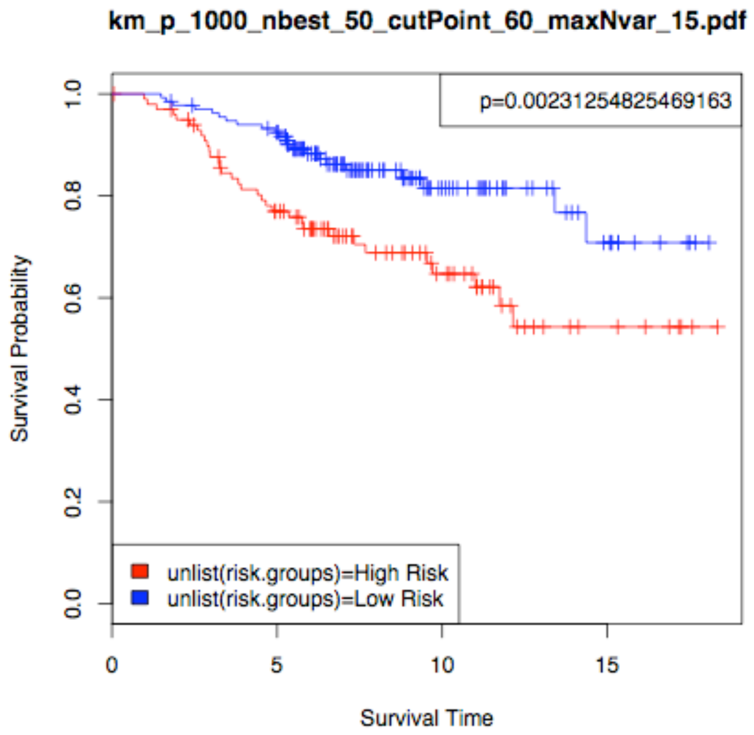

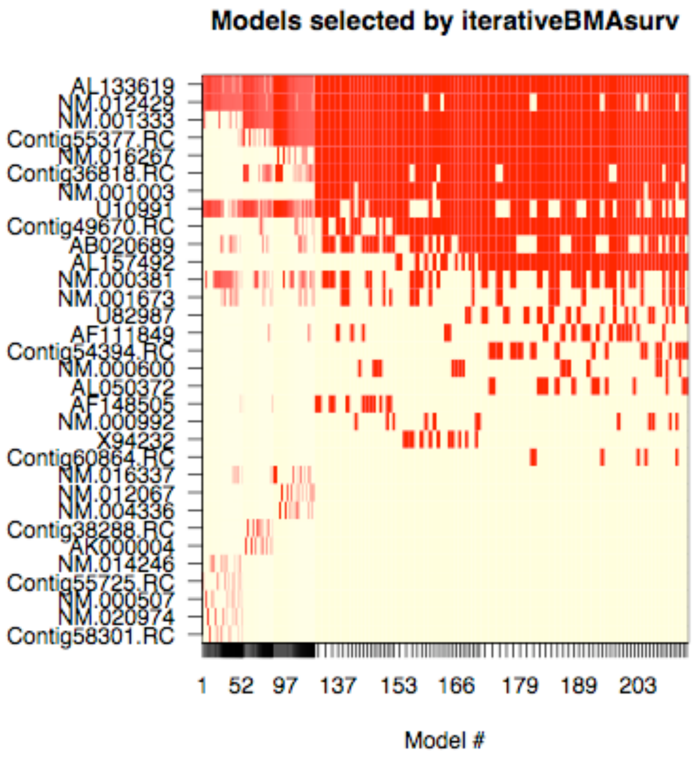

Breast cancer data n=234 with keepRmModels=T, top 5 genes

selected genes = 5
selected models = 7
Risk Table:

	cens.vec.test	
	0	1
High Risk	72	33
Low Risk	107	22

chisq from the log-rank test = 6.75414967612084
pvalue from the log-rank test = 0.00935299079366747

gene name	posterior probability	univariate rank
NM_012429	100.00%	1
AL133619	100.00%	2
NM_016267	29.83%	5
NM_001333	18.81%	4
Contig55377_RC	16.64%	3

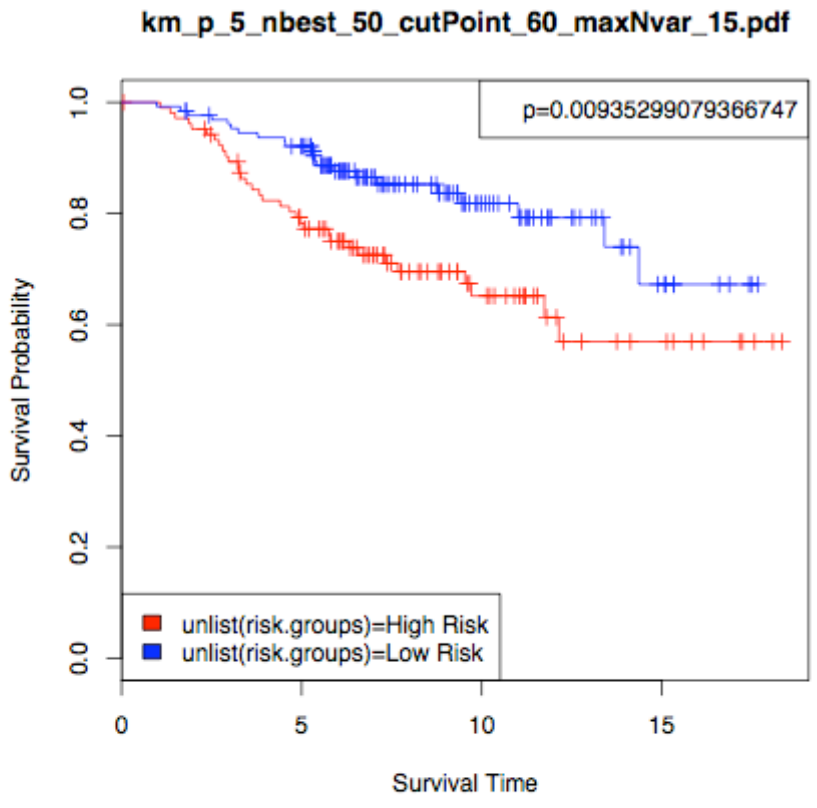

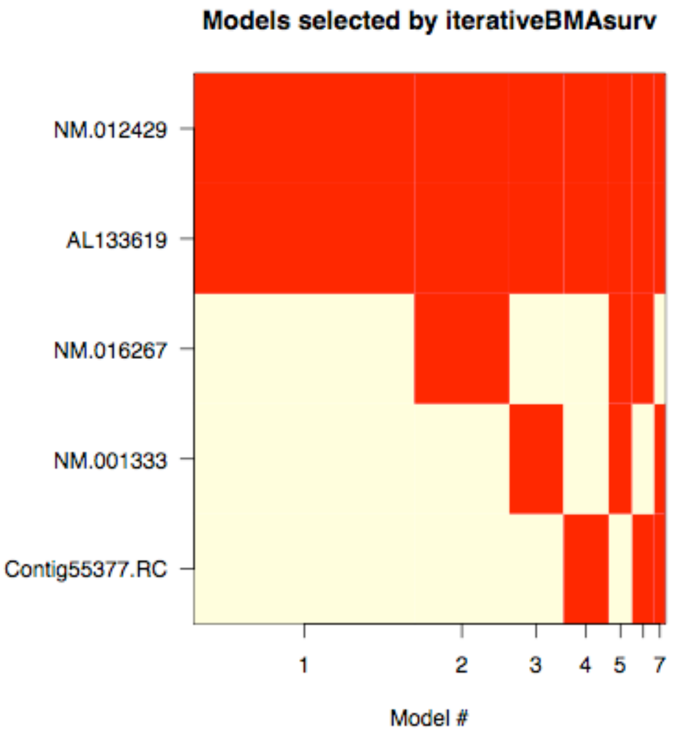

Breast cacner data n=295 with keepRmModels=T
selected genes = 32
selected models = 217
Risk Table:

	cens.vec.test	
	0	1
High Risk	73	52
Low Risk	143	27

chisq from the log-rank test = 28.3982871874793
pvalue from the log-rank test = 9.87526407314476e-08

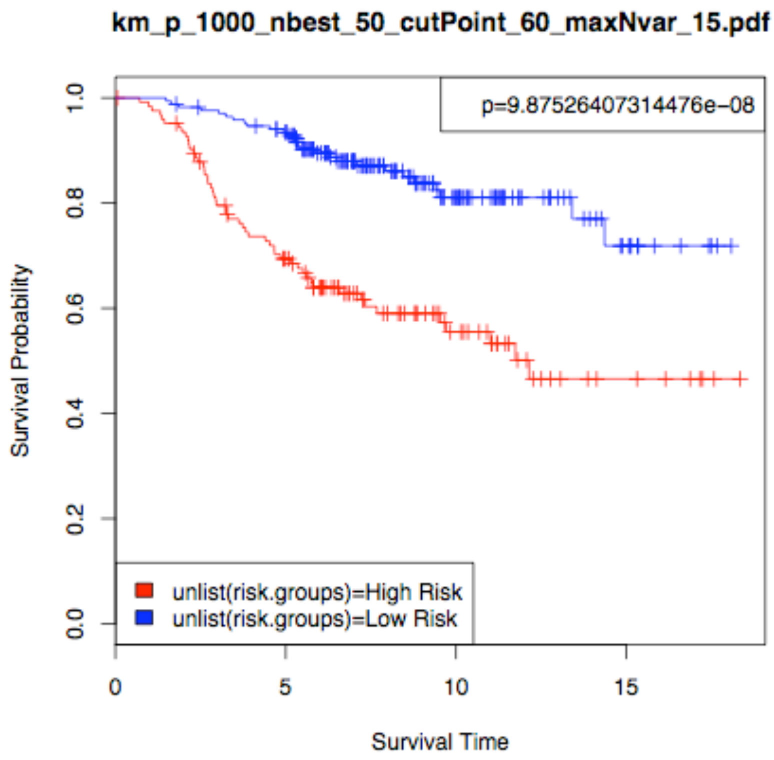

<u>Breast cacner data n=295 with keepRmModels=T, top 5 genes</u>

\# selected genes = 5

\# selected models = 7

Risk Table:

	cens.vec.test	
	0	1
High Risk	79	51
Low Risk	137	28

chisq from the log-rank test = 20.2410800318559

pvalue from the log-rank test = 6.82710372179596e-06

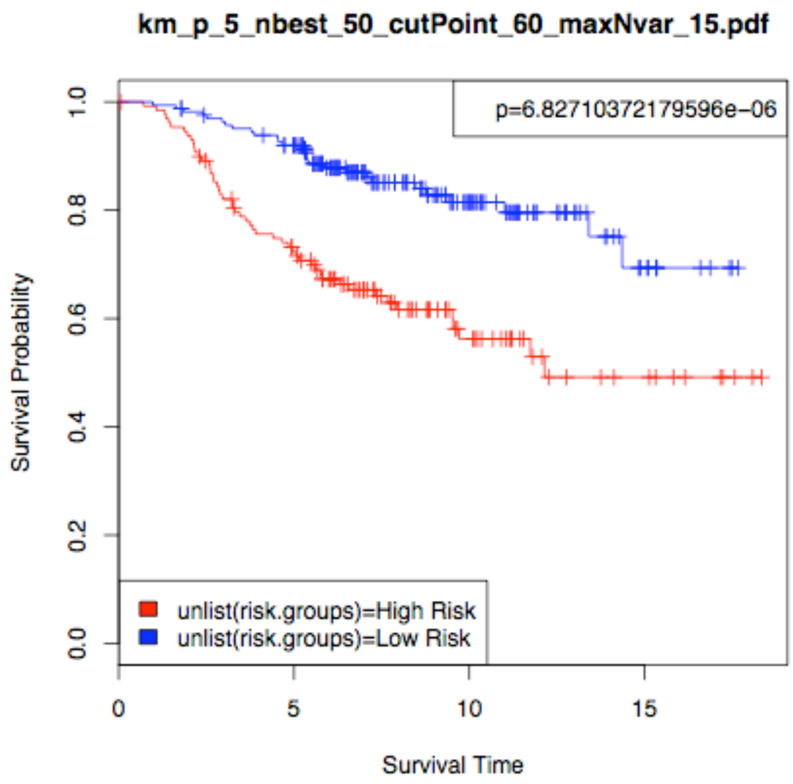

DLBCL data with keepRmModels=T
selected genes = 38
selected models = 55
Risk Table:

	cens.vec.test	
	0	1
High Risk	8	23
Low Risk	22	27

chisq from the log-rank test = 4.03808799493837
pvalue from the log-rank test = 0.0444841941309219

gene name	posterior probability	univariate rank
X31981	100.00%	1
X32679	100.00%	4
X27415	93.47%	16
X17482	87.24%	31
X28197	84.86%	6
X27774	74.15%	24
X31471	66.68%	33
X27267	62.75%	30
X34488	57.86%	45

X24452	49.84%	41
X33014	33.05%	2
X30258	31.89%	59
X24376	22.88%	49
X33310	16.70%	42
X30040	15.53%	47
X31242	11.22%	7
X27766	10.99%	43
X17154	10.99%	61
X24631	10.99%	62
X30945	6.60%	52
X26940	6.46%	53
X31806	6.10%	57
X27731	5.75%	69
X27182	5.56%	63
X30016	4.07%	10
X30765	3.84%	28
X34785	3.45%	8
X24394	2.66%	20
X19255	1.50%	12
X24396	1.42%	23
X34015	1.40%	26
X19373	1.39%	14
X27573	1.37%	15
X27585	1.30%	18
X34500	1.29%	9
X17591	1.17%	19
X30742	0.89%	21
X17316	0.45%	17

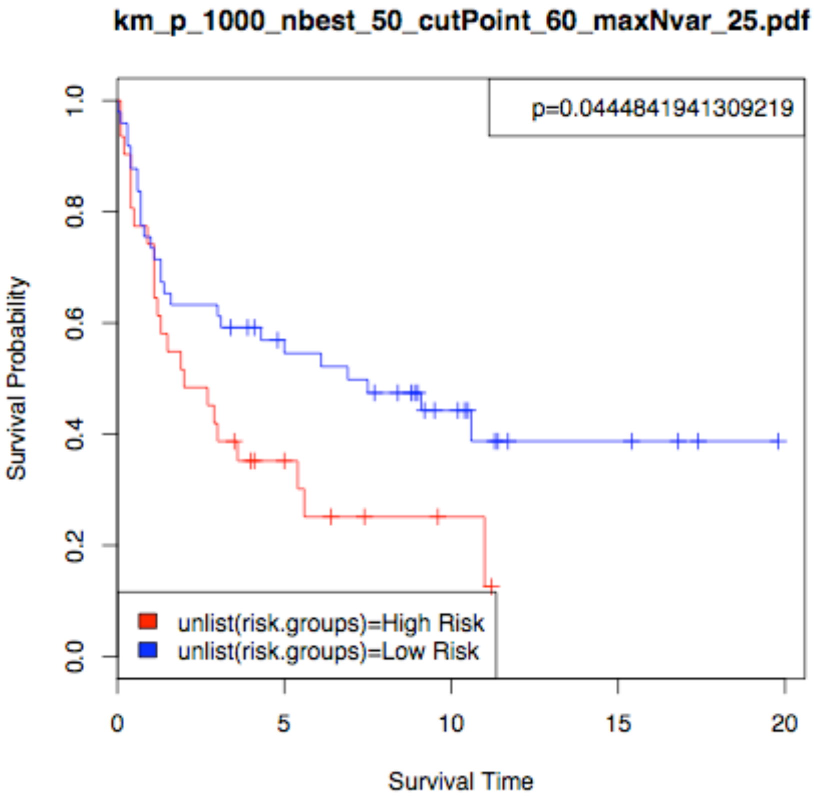
km_p_1000_nbest_50_cutPoint_60_maxNvar_25.pdf
p=0.0444841941309219
Survival Probability
1.0
0.8
0.6
0.4
0.2
0.0
unlist(risk.groups)=High Risk
unlist(risk.groups)=Low Risk
0
5
10
15
20
Survival Time

Models selected by iterativeBMAsurv

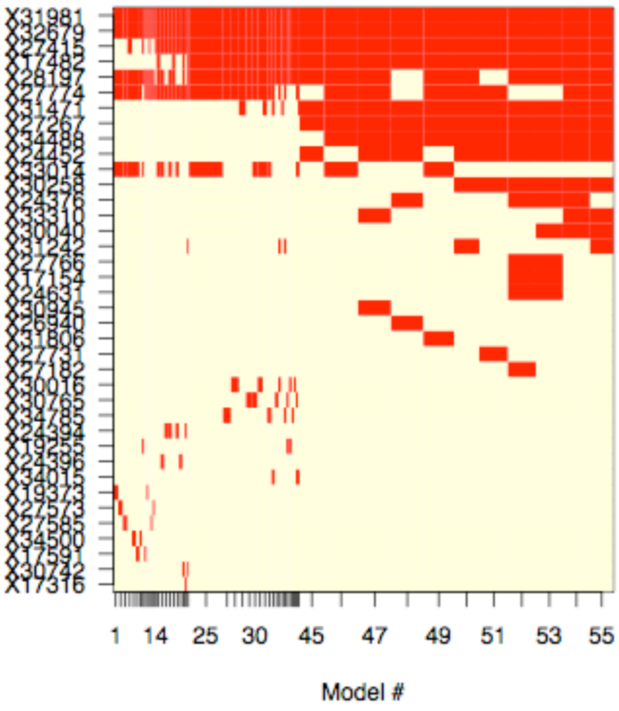

Supplement: Additional file 1 — Supplementary Materials. This document contains supplementary tables, figures and method. [file 1471-2105-10-72-S1.pdf]
